# Graphene oxide electrocatalyst on MnO_2_ air cathode as an efficient electron pump for enhanced oxygen reduction in alkaline solution

**DOI:** 10.1038/srep09108

**Published:** 2015-03-13

**Authors:** Wan Jeffrey Basirun, Mehran Sookhakian, Saeid Baradaran, Zulkarnain Endut, Mohammad Reza Mahmoudian, Mehdi Ebadi, Ramin Yousefi, Hanieh Ghadimi, Sohail Ahmed

**Affiliations:** 1 Department of Chemistry, University Malaya, Kuala Lumpur 50603, Malaysia; 2 Institute of Nanotechnology & Catalysis (NanoCat), University Malaya, 50603 Kuala Lumpur, Malaysia; 3 Department of Chemistry, Shahid Sherafat, University of Farhangian, 15916, Tehran, Iran; 4 Department of Chemistry, Faculty of Sciences, Islamic Azad University, Gorgan, 49147-39975 Iran; 5 Department of Physics, Masjed-Soleiman Branch, Islamic Azad University, Masjed-Soleiman, Iran; 6 Center of Foundation Studies, Faculty of Science, Universiti Putra Malaysia, 43400 UPM Serdang, Selangor, Malaysia

## Abstract

Graphene oxide (GO) was deposited on the surface of a MnO_2_ air cathode by thermal evaporation at 50°C from a GO colloidal suspension. Fourier transformed infrared spectroscopy and field emission scanning electron microscopy confirmed the presence of GO on the MnO_2_ air cathode (GO-MnO_2_). Voltammetry and chrono-amperometry showed increased currents for the oxygen reduction reaction (ORR) in 6 M KOH solution for GO-MnO_2_ compared to the MnO_2_ cathode. The GO-MnO_2_ was used as an air cathode in an alkaline tin-air cell and produced a maximum power density of 13 mW cm^−2^, in contrast to MnO_2_, which produced a maximum power density of 9.2 mW cm^−2^. The electrochemical impedance spectroscopy results suggest that the chemical step for the ORR is the rate determining step, as proposed earlier by different researchers. It is suggested that the presence of GO and electrochemically reduced graphene oxide (ERGO) on the MnO_2_ surface are responsible for the increased rate of this step, whereby GO and ERGO accelerate the process of electron donation to the MnO_2_ and to adsorbed oxygen atoms.

The oxygen reduction reaction (ORR) is one of the most widely studied reactions, particularly for fuel cell and metal-air battery applications. Due to the slow kinetics of the ORR, several materials have been developed as electro-catalysts to accelerate the reaction. Different types of graphene, such as graphene nanosheets^1^ and porous graphene^2^ are good electro-catalysts for ORR in lithium-air cells. Graphene-based materials such as nitrogen doped graphene^3^ and graphene-based Fe-N-C materials^4^ are also important electro-catalyst for the ORR. The most widely used electro-catalyst for the ORR is MnO_2_ due to its low cost and high electro-catalytic activity^5^,^6^. In alkaline solution, the ORR proceeds with a 4 electron reduction pathway for MnO_2_^5^,^6^,^7^ and graphene^4^,^8^. The mechanism of the ORR on electro-catalytic MnO_2_ has been studied extensively but is not fully understood. Generally, the ORR in alkaline solutions involves a two-step process and can be given by eq. 1 and eq. 2^5^,^6^,^7^,^9^:
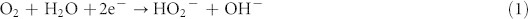

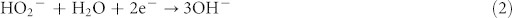
The combination of eq. 1 and eq. 2 gives an overall 4 electron reduction for the ORR in alkaline solution.

A detailed mechanism for the first step (eq. 1) on MnO_2_ in alkaline medium has been proposed^7^,^9^:
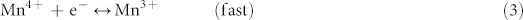

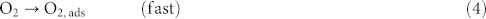

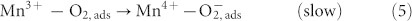

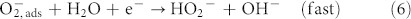
O_2, ads_ refers to adsorbed oxygen molecules on the MnO_2_ surface. The slowest step in the whole process is eq. 5, where electrons received by MnO_2_ in eq. 3 are excited and donated to the adsorbed oxygen molecule O_2, ads_. Although graphene materials are also good electro-catalyst for the ORR, the superior performance of MnO_2_ remains unchallenged by any pure graphene materials. Recently Yang et al.^10^ used MnO_2_-graphene nanosheets as the air cathode in lithium-air cells and obtained a discharge capacity much higher than pure graphene nanosheets (GNS) catalyst. On the other hand, pure MnO_2_ has lower performance toward the ORR, compared to composites of MnO_2_ and graphene materials. Wen et al.^11^ used MnO_2_-graphene nanosheet composites as the air cathode in microbial fuel cells (MFC) and obtained a power density higher than a pure MnO_2_ catalyst. Gnanakumar et al.^12^ used nanotubular MnO_2_-GO composites as the air cathode in MFC and obtained a power density higher than the pure MnO_2_ nanorods and MnO_2_ nanotubes. Qian et al.^13^ also obtained larger voltammetric currents for ORR with MnO_2_/reduced graphene oxide (RGO) composites compared to pure RGO in alkaline solution and also showed that the MnO_2_-RGO composite proceeds with a 4 electron reduction pathway for the ORR. In this work, the formation of stacked graphene oxide (GO) layers on a MnO_2_ air cathode and their electro-catalytic performance in an alkaline tin-air cell are investigated.

## Results

**Figure 1:**
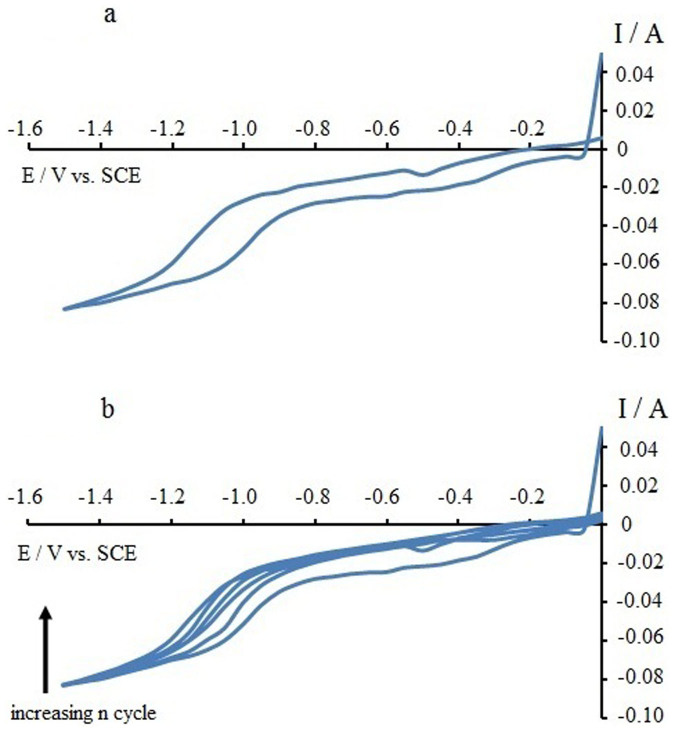
The CV for the electrochemical reduction of GO onto MnO_2_ substrate, (a) first cycle (b) all four cycles.

**Figure 2:**
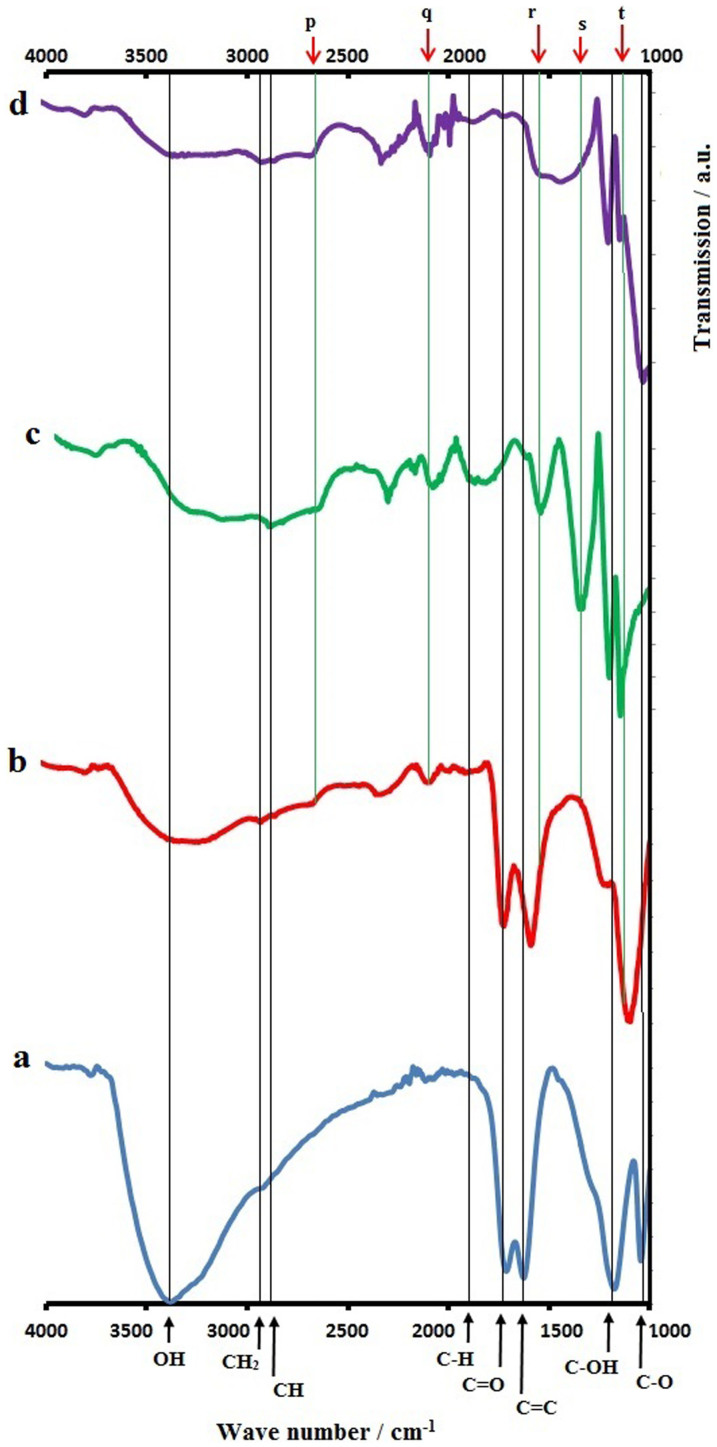
FTIR of (a) GO (b) GO-MnO_2_ before discharge, dried (c) GO-MnO_2_ after discharge, not dried (d) ERGO-MnO_2_.

Figure 1a shows the CV for the first cycle of the electrochemical reduction of GO suspension in 0.1 M phosphate buffer solution, on the MnO_2_ substrate. The first anodic wave at around −0.4 V to −0.8 V and the second wave beyond −1.0 V are attributed to the redox pair of oxygen-containing groups on the graphene plane^14^,^15^. Figure 1b shows all four cycles for the reduction of GO to ERGO on the MnO_2_ substrate. It can be observed that the currents became smaller with increasing cycle numbers. These results are also consistent with previous results on the electro-reduction of GO onto polypyrrole nano-fibers^16^. Figure 2a shows the FTIR spectrum of the synthesized GO. All of the characteristic peaks for OH, C = O, C = C, C-OH and C-O are present at approximately 3400, 1730, 1630, 1230 and 1070 cm^−1^, respectively, in the GO spectrum. Figure 2b is the FTIR spectrum of the GO-MnO_2_ air cathode, where the peaks for OH, C = O, C = C and C-OH are still present. The peaks at 2950 and 2870 cm^−1^ are due to the CH_2_ and CH vibrations, respectively, from the carbon black additive in the MnO_2_ air cathode^17^. Due to the thermal evaporation of the GO-MnO_2_ air cathode, the OH peak at approximately 3400 cm^−1^ becomes smaller compared to the C = O and C = C peaks in the FTIR spectrum in Fig. 2b. In Figs. 2b and 2c, the bands at *p*, *q*, *r, s* and *t* are due to the MnO_2_ substrate layer. The band at approximately 2700 cm^−1^ (*p*) is due to the fundamental OH stretching with an OH^...^O length of approximately 2.60 Å in the MnO structure^18^. The peak at approximately 2100 cm^−1^ (*q*) is due to the combination of the OH stretching mode at 2700 cm^−1^ and the excited lattice mode at approximately 600 cm^−1^, where 2700 − 600 = 2100 cm^−1^, and is very close to previous reports^19^,^20^. The peaks at approximately 1550 and 1360 cm^−1^ (*r* and *s*, respectively) are attributed to the interaction of Mn with surrounding species such as OH and O^21^. The peak at approximately 1200 cm^−1^ (*t*) is due to the vibration of the hydrated MnO_2_^21^. The peaks at approximately 1550 and 1360 cm^−1^ (*r* and *s*, respectively), which are due to the interaction of Mn with OH, are evident in Fig. 2c (GO-MnO_2_, after discharge, not dried) compared to Fig. 2b (GO-MnO_2_, before discharge, dried).

Figure 2c is the FTIR spectrum of the GO-MnO_2_ after cell discharge, not dried. Figure 2c shows that the C = O peak height, at approximately 1730 cm^−1^, decreases in the FTIR spectrum of GO-MnO_2_ after discharge, compared to Fig. 2b. In Fig. 2b, the C = O peak height at 1730 cm^−1^ is larger than the C-OH peak height at 1230 cm^−1^. However, in Fig. 2c, the C-OH peak height is larger than the C = O peak, which also suggests that the carbonyl groups were electrochemically reduced during the discharge of the GO-MnO_2_ air cathode. Due to the close proximity with the *r* peak, it is unclear whether the C = C peak at approximately 1630 cm^−1^ is slightly decreased in Fig. 2c (GO-MnO_2_, after discharge, not dried), compared to Fig. 2b.

The CH_2_ and CH vibrations at 2950 and 2870 cm^−1^, respectively, in Fig. 2b have lower peak heights compared to C = O at 1730 cm^−1^. However, in Fig. 2c, the CH_2_ and CH vibrations have larger peak heights compared to the C = O peak. This difference could be due to the reduction of the peripheral COOH, (the Lerf-Klinowskii model^22^) to CH_2_OH during cell discharge. The bands approximately 1800–2000 cm^−1^ are attributed to the coupling and overtone bands of the aromatic C-H out of plane bending vibration modes^23^,^24^. These bands at approximately 1800–2000 cm^−1^ are also present in Fig. 2b and are attributed to the carbon black additive in the MnO_2_^17^. The XPS spectrum of the ERGO electrodeposited on MnO_2_ (ERGO-MnO_2_) is shown in Fig. 2d, and resembles the XPS of the GO-MnO_2_ after discharge (Fig. 2c)

The FESEM images (supplementary information Figure SI-1) of the MnO_2_ and GO-MnO_2_ and ERGO-MnO_2_ air cathode surfaces show that the GO and ERGO flat sheets are stacked on one another on the MnO_2_ substrate, where GO has only partial surface coverage on the MnO_2_ substrate. It can be observed that GO has sheet morphology, whereas MnO_2_ has grain morphology due to the preparation methods described in the experimental section.

### X-ray photoelectron spectroscopy

The XPS spectra of the C 1s binding energy for GO-MnO_2_ before discharge, dried, and GO-MnO_2_ after discharge, not dried are shown in the supporting information (SI-2a and SI-2b). The binding energies at 284.5 eV and 284.6 eV (SI-2a and SI-2b, respectively), are attributed to C-C^25^,^26^,^27^,^28^,^29^. The FTIR in Fig. 2c shows the appearance of CH_2_ and CH groups at approximately 2950 cm^−1^ and 2870 cm^−1^, respectively, and the binding energy of the C-H (SI-2b, GO- MnO_2_ after discharge) are also assigned close to the C-C at 284.5–285 eV in the C 1s spectrum^25^. The binding energy at 286.4 eV and 286.1 eV (SI-2a and SI-2b, respectively) are attributed to C-OH^26^,^27^,^28^, which has the same binding energy as C-O-C^28^,^30^. The binding energy at 288.2 eV (SI-2a and SI-2b) is attributed to C = O^29^, while the binding energy at 289.2 eV (SI-2a) is attributed to O = C-OH^28^,^29^. It can be observed (SI-2b) that the peak height and the area under the curve of the C-O peak is increased slightly compared to the C = O peak, which suggests that the electrochemical reduction of C = O to C-O took place during cell discharge, as confirmed by the FTIR result in Fig. 2c. The XPS results also suggest that not all C = O has been reduced to C-O during cell discharge and that some of the GO remains on the surface of the MnO_2_. This finding suggests that electro-reduction of the GO to ERGO occur only on the GO surface, consistent with previous reports^31^. A similar trend for the decrease in the peak height and the area under the curve of the C = O peak, and the increase of the peak height and area under the curve of the C-O peak, was observed when GO was reduced with a mixture of argon and hydrogen gases at 200°C, 500°C and 1000°C in a tube furnace^32^.

The O 1s spectra for GO-MnO_2_ before and after discharge are shown in supporting information (SI-2c and SI-2d), respectively. The binding energies of oxygen bonded Mn atoms (Mn-O and Mn-OH) have been reported to be 530.8 eV and 530.9 eV^33^,^34^. Therefore, it can be observed that the binding energy of Mn-O in GO-MnO_2_ before and after discharge, (SI-2c and SI-2d respectively) is 531.1 eV. The binding energy of C-O has been reported at 533 eV, while the C = O binding energy, which also has a contribution from O = C-OH^32^, is 530.6 eV. Thus, the binding energy of C = O at 530.6 eV (SI-2c) together with the binding energy of C-OH at 532.6 eV and at 533 eV (SI-2c and SI-2d, respectively), are in close agreement with previous reports. It can be observed that the C = O peak at 530.5 eV diminishes (SI-2d) when the GO-MnO_2_ was discharged as the air cathode, which is due to reduction of the C = O to C-OH. A similar result was also reported when GO was treated at 1000°C with a mixture of argon and hydrogen gases, where a peak binding energy of C = O disappeared due to reduction by hydrogen gas, while the peak binding energy at 533 eV, attributed to C-O, remained in the O 1s spectrum^32^. When water is introduced to the MnO_2_, the binding energy for the absorbed water (O-H_2_) is 532.5 eV in the O 1s spectrum^33^,^34^. Therefore the peak at 533 eV in the GO-MnO_2_ after discharge not dried spectrum (SI-2d) is attributed to the absorbed water molecules. The peak height and area under the curve for the C-OH (532.6 eV) peak are larger compared to the corresponding Mn-O (531.1 eV) in SI-2c. This difference is due to the absence of water in the GO-MnO_2_ before discharge, dried cathode. However, the peak height and area under the curve at 533 eV and 531.1 eV (SI-2d) of the C-OH and Mn-O, respectively, are almost identical, which is due to the interaction of absorbed water with Mn (Mn-OH_2_), in the GO-MnO_2_ after discharge, not dried sample. The XPS spectra of the C 1s and O 1s (SI-2e and SI-2f) of the ERGO-MnO_2_ prepared by electrodeposition shows similarities to the C 1s and O 1s spectra of the GO-MnO_2_ after discharge (SI-2b and SI-2d, respectively).

### Voltammetry, chrono-amperometry and tin-air cell discharge

The working electrodes (WE) were attached to a cell holder in which the Teflon membrane was exposed to oxygen diffusion from air, while the MnO_2_ electro-catalytic surface was exposed to the KOH solution. Voltammetry results from the MnO_2_, GO-MnO_2_ and ERGO-MnO_2_ electrodes in 6 M KOH solution at 5 mV s^−1^ (supporting information Figure SI-3a and SI-4a) shows that the ORR current of the GO-MnO_2_ and ERGO-MnO_2_ cathodes are higher compared to that of the MnO_2_ cathode at all potentials. Chrono-amperometry at −0.9 V and the steady-state currents from chrono-amperometry at potentials −0.5, −0.6, −0.7, −0.8, −0.9 and −1.0 V, show that the currents for the GO-MnO_2_ and ERGO-MnO_2_ cathodes are higher compared to the MnO_2_ at all potentials (supporting information SI-3b, SI-3c, SI-4b and SI-4c).

The MnO_2_, GO-MnO_2_ and ERGO-MnO_2_ air cathodes were assembled in an undivided tin-air cell in 6 M KOH. The reactions at the positive and negative electrodes are as follows:
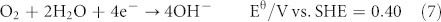

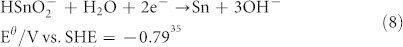


The cell notation for the alkaline tin-air cells can be represented as “Sn|6 M KOH|MnO_2_” and “Sn|6 M KOH|GO-MnO_2_”. The Sn-MnO_2_ and Sn-MnO_2_/GO cells are discharged until complete dissolution of the tin negative electrode is reached. The OCP measurements for both cells yielded an average value of 1.20 V, which is close to the standard values calculated in eq. 7 and eq. 8. The chrono-potentiometry plots (SI-5a and SI-5a) at discharge currents of 3, 5, 10, 15, 20, 25 and 30 mA cm^−2^ for the Sn-MnO_2_ and Sn-MnO_2_/GO cells, respectively show that the GO-MnO_2_ air cathode gives higher potentials compared to MnO_2_ for all discharge currents (SI-5c). From the power density plots (P vs. I) for the tin-air cells with the MnO_2_ and GO-MnO_2_ air cathodes (SI-5d), it can be observed that the GO-MnO_2_ produces higher power density compared to the MnO_2_ air cathode. The maximum power density of the Sn-MnO_2_ cell is 9.2 mW cm^−2^, while the maximum power density of the Sn-GO/MnO_2_ cell is more than 13 mW cm^−2^ (SI-5d), and both are higher than the power density of 6.8 mW cm^−2^ obtained from Zn-air cells^36^. The current efficiencies that are calculated from weight loss measurements of the tin negative electrode using the Faraday equation (*It = mnF*, *n = 2*) and the discharge profiles (SI-4) are close to 100% for all discharge currents. The charge densities for the dissolution of tin are in the range of 446–449 mAh g^−1^ for all current densities, which is in accordance with the dissolution of Sn (atomic weight 119) to Sn (II) and is higher than the value of 360 mAh g^−1^ that has been reported for Zn-air^37^. The chrono-potentiometry and power density plots of ERGO-MnO_2_ (SI-6) also gave higher potentials and power density compared to the bare MnO_2_ air cathode. The result of this work and previous works in MFC using MnO_2_ graphene composite air cathodes are summarized in the supplementary information (Table SI-1). From the results of other researchers^11^,^12^ in the supporting information (Table SI-1) the increase of power density compared to the bulk MnO_2_ cathode is 1.42 and 1.82 respectively, while in this work, the increase of power density of the GO-MnO_2_ and ERGO-MnO_2_ compared to the pure MnO_2_ cathode, is 1.40 and 1.46 respectively(Table SI-1). The results of other researchers^11^,^12^ reports the bulk modification of the MnO_2_ with graphene and GO respectively, while this work reports the surface modification of the MnO_2_ with GO and ERGO.

### Electrochemical impedance spectroscopy

EIS was performed on the MnO_2_, GO-MnO_2_ and ERGO-MnO_2_ cathodes in 6 M KOH solution. A two-electrode configuration was used in these EIS experiments, where the MnO_2_, ERGO-MnO_2_, GO-MnO_2_ air cathodes before and after discharge were the WE, while a Hg/HgO electrode in 6M KOH solution was the RE and CE. The Nyquist plots of all four air cathodes are shown in the supplementary information (Figure SI-7). The Nyquist plots show one semi-circle at higher frequencies and a Warburg element at lower frequencies. The *R_1_(Q[R_2_W_1_])* equivalent circuit model was found to accurately fit the experimental data, where an excellent agreement was obtained between the experimental data and the simulation of the equivalent circuit model, and the chi-squared (*x*^2^) value was minimized to 10^−4^. The *R_1_(Q[R_2_W_1_])* equivalent circuit diagrams of the “MnO_2_/6 M KOH/Hg-HgO”, “ERGO-MnO_2_/6 M KOH/Hg-HgO” and the “GO-MnO_2_/6 M KOH/Hg-HgO” systems are similar to the circuit diagram from previous reports^11^,^12^.

According to the EIS measurements of the ERGO-MnO_2_, GO-MnO_2_ and MnO_2_ cathodes in 6 M KOH, *R_2_* is the charge transfer resistance and *Q_1_* is the CPE for the ORR. The diameter of semicircle represents the charge transfer resistance (Rct) across the electrode-electrolyte interface^11^. The computer simulations of the EIS experiments indicate that the *R_2_* (Rct) of the MnO_2_, GO-MnO_2_ air cathodes before discharge and after discharge, and ERGO-MnO_2_ are 310.0 Ohm, 309.0 Ohm, 294.5 Ohm and 294.0 Ohm, respectively. The results of this work and previous report in MFC are shown in the supplementary information (Table SI-2). The charge transfer resistances (Rct) observed for the electrodes fall in the order of MnO_2_ nanotubes > Pt/C > MnO_2_ nanotubes/GO composite^12^. The smaller Rct of the prepared MnO_2_/GO composite represents an enhanced reaction rate kinetics with a decreased charge transfer resistance, which is ascribed to the good contact of MnO_2_ nanotubes with the carbon support GO and high electrical conductivity of the composite material^12^. The result of this work is in good agreement with previous reports^11^,^12^.

**Figure 3:**
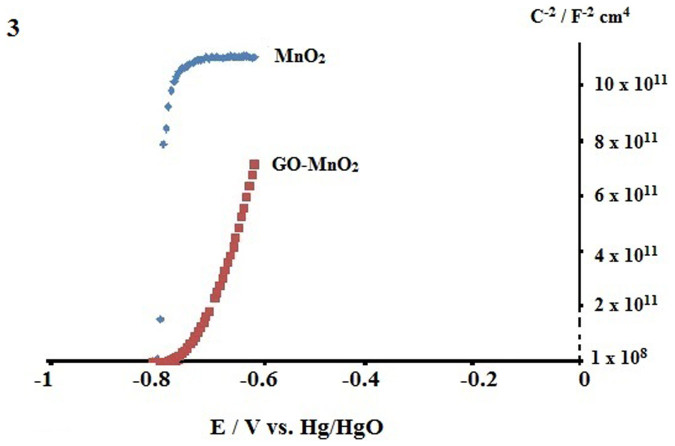
Mott-Schotkky plots of MnO_2_ and GO-MnO_2._

The Mott-Schottky plot of MnO_2_ and GO-MnO_2_ in 6M KOH is shown in Fig. 3. From the Mott-Schottky relation:
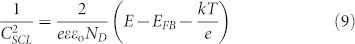


Where C_SCL_ is the capacitance of the space charge layer, e is the charge of electron, ε_o_ is permittivity of free space 8.85 × 10^−12^ F m^−1^, ε is dielectric constant of MnO_2_, E is applied potential, E_FB_ is flat-band potential, N_D_ is donor density, k is Boltzmann constant, T is Kelvin temperature. The plot of C^−2^ vs E/V for MnO_2_ shows a positive slope for both GO-MnO_2_ and MnO_2_ which is typical for an n-type semiconductor. From the slope of the linear part of the plot close to the flat-band potential (2/eεε_o_N_D_), shows that the slope for GO-MnO_2_ is lower than MnO_2_. This suggests that the charge carrier (electron for n-type semiconductor) density is larger for the GO-MnO_2_ compared to MnO_2_.

## Discussion

From the FTIR spectra, it can be concluded that the GO on the MnO_2_ cathode surface was reduced to electrochemically reduced graphene oxide (ERGO) during cell discharge in 6M KOH, and this finding is in accordance with the electrochemical reduction of GO to ERGO in concentrated alkaline solution^31^. Tin-air discharge, LSV and chrono-amperometry results confirm the increased electro-catalytic effect of the GO-MnO_2_ cathode toward the ORR compared to the MnO_2_ without the GO.

In the EIS results, the smaller *R_2_* (Rct) of the GO-MnO_2_ air cathode after discharge is due to the reduction of GO to ERGO on the MnO_2_ surface during cell discharge. Therefore, the presence of ERGO is responsible for the lower *R_2_* of the GO-MnO_2_ air cathode after discharge. It can be observed that ERGO has lower interfacial charge transfer resistance compared to GO due to the higher electrical conductivity of ERGO compared to GO^38^, which is consistent with previous work^39^. Therefore, during cell discharge, some of the GO is reduced to ERGO, and this decreases the interfacial charge transfer resistance of the GO-MnO_2_ cathode after discharge. The EIS simulation results show only a small difference between the *R_2_* of the MnO_2_ and GO-MnO_2_ air cathodes before discharge, while the GO-MnO_2_ air cathode after discharge has the smallest *R_2_* value. However, voltammetry, chrono-amperometry and the tin-air cell discharge results clearly show higher currents and higher power densities for the GO-MnO_2_ compared to the MnO_2_ air cathode. The ORR mechanisms on the MnO_2_ electro-catalyst in alkaline solution are given in eq. 3 to eq. 6. The charge transfer resistance (*R_2_*) is the resistance against the interfacial electron transfer process that occurs across the air cathode-KOH electrolyte interface for the ORR. Therefore, eq. 3 and eq. 6 are related to the rate of the electron transfer process, and *R_2_* depends only on eq. 3 and eq. 6. However, the electron transfer steps in eq. 3 and eq. 6 are fast reactions and thus are not rate determining steps in the ORR. Hence, the *R_2_* values of the MnO_2_ and GO-MnO_2_ air cathodes before and after discharge cannot explain the increased currents and power densities for the GO-MnO_2_ air cathode.

**Figure 4:**
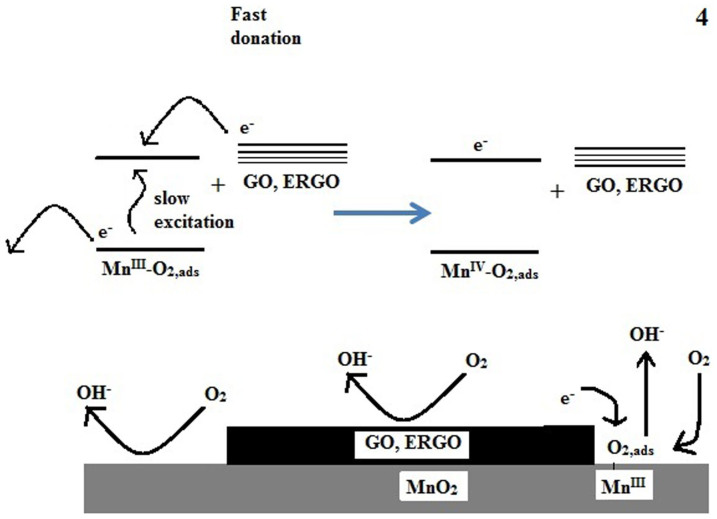
Diagram of the electron donating effect of GO and ERGO on MnO_2_ adsorbed oxygen Mn^3+^-O_2,ads_.

The overall current for the ORR depends on the reaction mechanisms proposed in eq. 3 to eq. 6. The chemical step in eq. 5, where the electron is excited from the valence band to the conduction band of the MnO_2_ electro-catalyst and is then donated to the adsorbed oxygen molecule, is the slowest step. This step has been proposed to be the rate determining step^7^,^9^, and therefore, this step controls the overall current of the ORR. An increase in electrical conductivity in graphene composite materials has been reported^40^, which is due to a lowering of the band-gap of the composite materials in the presence of graphene. The presence of ERGO on the surface of polypyrrole nanofibers, which increases the electrical conductivity of the nanofibers by decreasing the band-gap of polypyrrole, has been proposed^16^. There are two possible reasons for the increased currents and power densities of the GO-MnO_2_ air cathode. First, the GO and ERGO are efficient electro-catalysts for the ORR, and their presence on the MnO_2_ surface provides a high surface area for the ORR to proceed. Second, the electron rich GO and ERGO on the MnO_2_ surface increase the rate determining step of the ORR. The increased electro-catalytic effect of the GO-MnO_2_ cathode for the ORR, is due to enhanced electron donation from the MnO_2_ electro-catalyst to the adsorbed oxygen molecule in the rate determining step (eq. 5). Given the presence of electron rich GO and ERGO on the surface of MnO_2_, electrons are transferred efficiently from the GO and ERGO to the conduction band of the MnO_2_, as shown in Fig. 4, thus increasing the rate of this step (eq. 5). Therefore, the presence of GO and ERGO on the MnO_2_ surface can result in faster electron donation in eq. 5, thus increasing the speed of the rate determining step of the ORR. From the Mott-Schottky results, the larger electron density in ERGO-MnO_2_ is responsible for the larger electron donation effect in the rate determining step for the ORR. Thus the presence of ERGO acts like an electron pump for MnO_2_ to accelerate the ORR.

Furthermore, at faster discharge rates, at currents higher than 15 mA cm^−2^, GO-MnO_2_ and provides higher power density than MnO_2_ (SI-5d and SI-6d). This effect is also due to the faster donation of electrons from the GO to MnO_2_ (Fig. 4) compared to the excitation process in eq. 5. The MnO_2_ air cathode provides lower power densities at higher discharge rates due to the slower electron excitation process and is thus unable to meet high demands at discharge rates that are faster than 15 mA cm^−2^. Given the presence of GO on the MnO_2_ surface, high demands at discharge rates that are faster than 15 mA cm^−2^ can be met by faster electron donation from the GO (and ERGO) to the MnO_2_ (Fig. 4), and thus, the GO-MnO_2_ and ERGO-MnO_2_ cathodes provide higher powder densities compared to the bare MnO_2_ cathode.

## Methods

### Synthesis

All chemicals were from Sigma Aldrich. The GO powder was prepared using a modified Hummers' method. The GO powder was dispersed in a beaker filled with distilled water and sonicated for 5 hours until the final concentration was 0.3 mg cm^−3^. The air cathode was catalytic MnO_2_ mixed with small amounts of carbon black to increase its conductivity. The powders were pressed onto a nickel mesh current collector, with one side attached to a Teflon membrane that was permeable to air but not to the electrolyte. The air cathode was placed in a jar with the MnO_2_ catalytic layer facing upwards. The GO dispersion was poured into a jar and evaporated overnight at 50°C in an oven (supplementary information Figure SI-8). In the electrodeposition of GO on the MnO_2_ cathode, GO powder (7 mg dm^−3^) was dispersed in 0.1 M phosphate buffer solution (K_2_HPO_4_ and KH_2_PO_4_) at pH 7.2 and sonicated for 5 hours. The electrodeposition of GO on the surface of MnO_2_ substrate was performed by cyclic voltammetry in a single compartment cell in 4 voltammetric scans. A saturated calomel electrode (SCE) was used as the reference, while a platinum foil with 2 cm^2^ surface area was the counter electrode. The potential range applied was from 0.0 to −1.5 V with the scan rate of 1 mV s^−1^. The Mott-Schotkky experiment was performed using a two electrode system, in 6M KOH, with the MnO_2_ and GO-MnO_2_ electrodes as the WE, and the Hg/HgO as the reference and counter electrodes, at a constant frequency of 1000 Hz.

### Characterization

The evaporated GO layers on the MnO_2_ air cathode (GO-MnO_2_) and electrodeposited ERGO- MnO_2_ were characterized using Fourier transformed infrared (FTIR) spectroscopy and field emission scanning electron microscopy (FESEM). The FTIR and FESEM instruments were Spectrum 400 and Quanta 200F, respectively. X-ray photoelectron spectroscopy (XPS) was conducted using a Kratos analytical axis ultra instrument with an Al K_α_ radiation source of 253.6 eV. Linear scan voltammetry (LSV), chrono-amperometry and electrochemical impedance spectroscopy (EIS) were performed using a potentiostat/galvanostat Autolab PGSTAT-302N from Ecochemie (Utrecht, Netherlands). LSV and chrono-amperometry were performed using a single compartment cell, with a mercury oxide (Hg/HgO) as the reference electrode (RE) and a graphite rod as the counter electrode (CE), while a two-electrode configuration was used in the EIS experiments. The EIS measurements were performed over a frequency range of 100 kHz to 10 mHz, with the acquisition of 10 points per decade, with a signal amplitude of 5 mV around the open circuit potential (OCP). The impedance spectra were analyzed by fitting the experimental results to equivalent circuits using a non-linear least-square fitting procedure with the chi-squared value minimized to 10^−4^. The OCP and tin-air cell discharge experiments were conducted using a Won-A-Tech WBCS 3000 (Korea Republic) battery cycler system. Tin metal foils with an area of 2 cm^2^ were used as the negative electrode in a 6 M KOH solution for the tin-air cell discharge experiments. The diameters of the MnO_2_ and GO-MnO_2_ positive electrodes were 0.9 cm in all experiments. The diagrams of the air cathode and the cell casing are described elsewhere^41^. The discharge capacity in mAh g^−1^ of the alkaline tin-air cell was measured from the chrono-potentiometry diagram and the weight loss of the tin negative electrode after cell discharge. All experiments were performed at room temperature, 27°C.

## Author Contributions

W.J.B. wrote the paper. S.B., M.S., R.Y., H.G. and S.M. prepared the GO and ERGO electrodes. M.R.M. and M.E. performed the EIS experiments. Z.E. ran the FTIR, XPS spectra and the other electrochemical experiments. All authors reviewed the revised manuscript and approved the submission.

## Supplementary Material

Supplementary Information Supporting Information
