# Supplementary material for: Graphene oxide electrocatalyst on MnO2 air cathode as an efficient electron pump for enhanced oxygen reduction in alkaline solution
Source: Sci Rep. 2015 Mar 13;5:9108. doi: 10.1038/srep09108 (PMC4357894; doi:10.1038/srep09108)
Supplement: Supplementary Information — Supporting Information [file srep09108-s1.pdf]

Supporting information

Graphene oxide electrocatalyst on MnO_2 air cathode as an efficient electron pump for enhanced oxygen reduction in alkaline solution.

Wan Jeffrey Basirun[2], Mehran Sookhakian[1], Saeid Baradaran[1], Zulkarnain Endut[6], Mohammad Reza Mahmoudian[3], Mehdi Ebadi[4], Ramin Yousefi[5], Hanieh Ghadimi[1], Sohail Ahmed[1].

[1]Department of Chemistry, University Malaya, Kuala Lumpur 50603, Malaysia.

[2]Institute of Nanotechnology & Catalysis (NanoCat), University Malaya, 50603 Kuala Lumpur, Malaysia.

[3]Department of Chemistry, Shahid Sherafat, University of Farhangian, 15916, Tehran, Iran.

[4]Department of Chemistry, Faculty of Sciences, Islamic Azad University, Gorgan, 49147-39975 Iran.

[5]Department of Physics, Masjed-Soleiman Branch, Islamic Azad University, Masjed-Soleiman, Iran.

[6]Center of Foundation Studies, Faculty of Science, Universiti Putra Malaysia, 43400 UPM Serdang, Selangor, Malaysia.

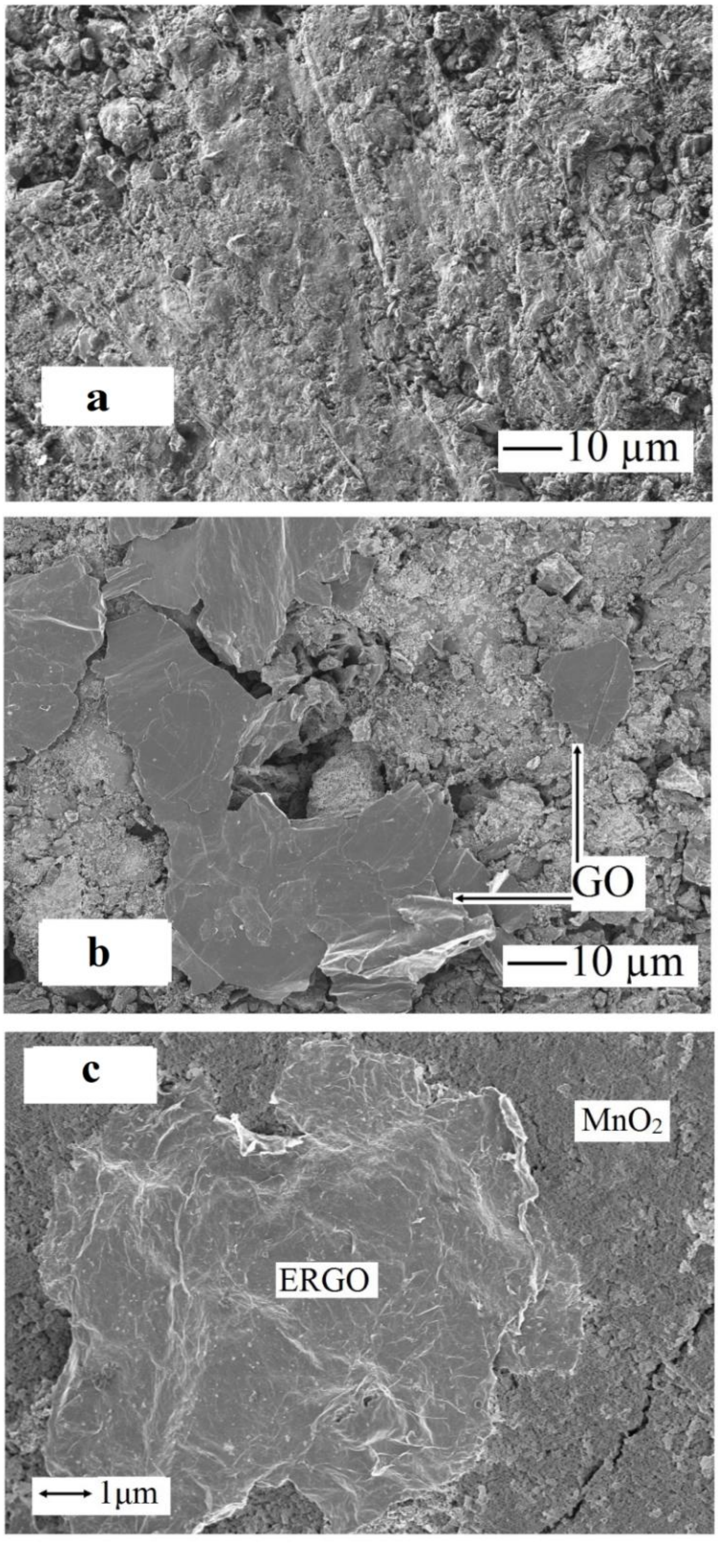

Figure SI-1. FESEM of (a) MnO_2 (b) GO-MnO_2 (c) ERGO-MnO_2.

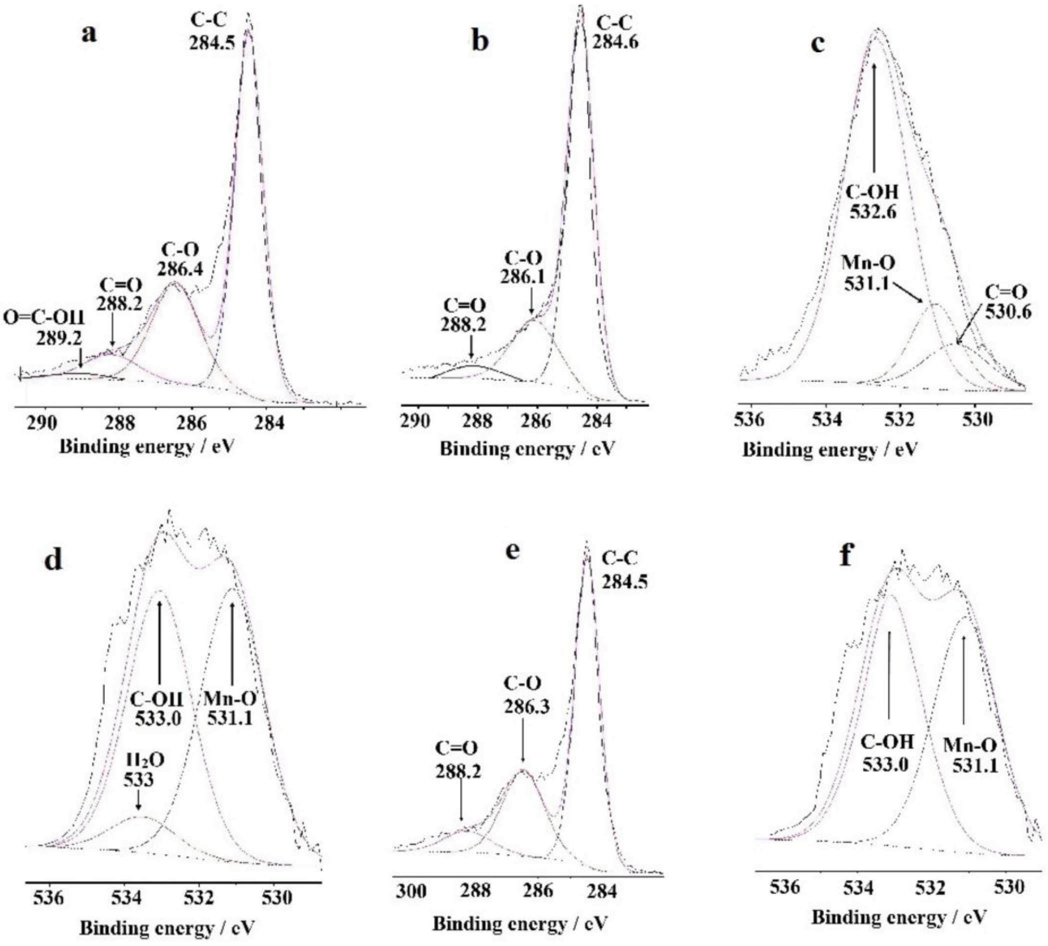

Figure SI-2. XPS spectra (a) C 1s of GO-MnO_2 before discharge dried (b) C 1s of GO-MnO_2 after discharge not dried (c) O 1s of GO-MnO_2 before discharge dried (d) O 1s of GO-MnO_2 after discharge not dried (e) C 1s of ERGO-MnO_2 (f) O 1s of ERGO-MnO_2.

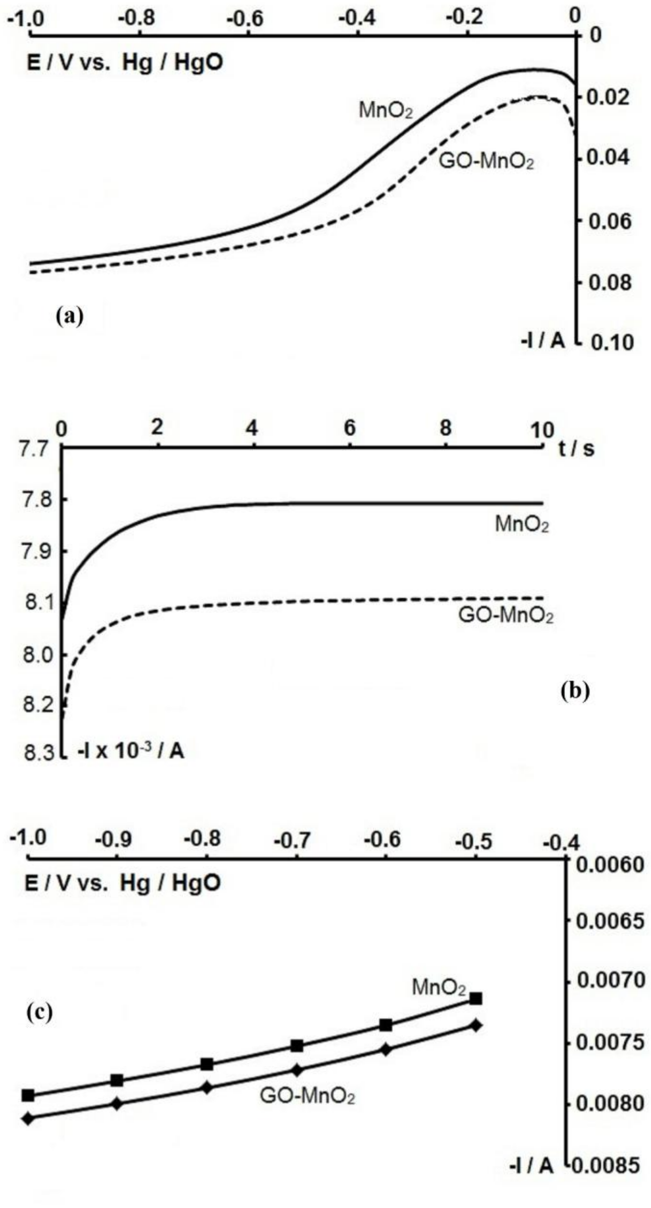

Figure SI-3. Electroanalytical results for MnO_2 and GO-MnO_2 (a) Voltammetry in 6M KOH solution at 5 mV s^{-1} (b) Chrono-amperometry at -0.9 V (c) Steady-state currents from chrono-amperometry at various potentials.

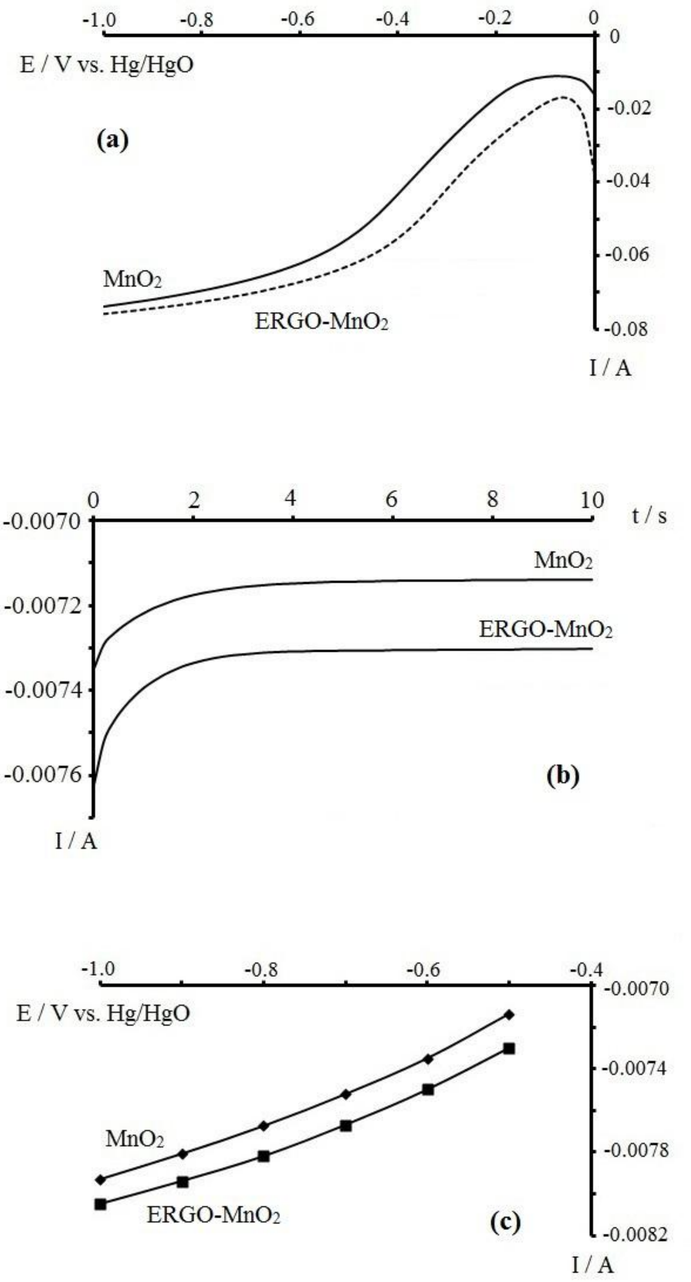

Figure SI-4. Electroanalytical results for MnO_2 and ERGO-MnO_2 (a) Voltammetry in 6M KOH solution at 5 mV s^{-1} (b) Chrono-amperometry at -0.9 V (c) Steady-state currents from chrono-amperometry at various potentials.

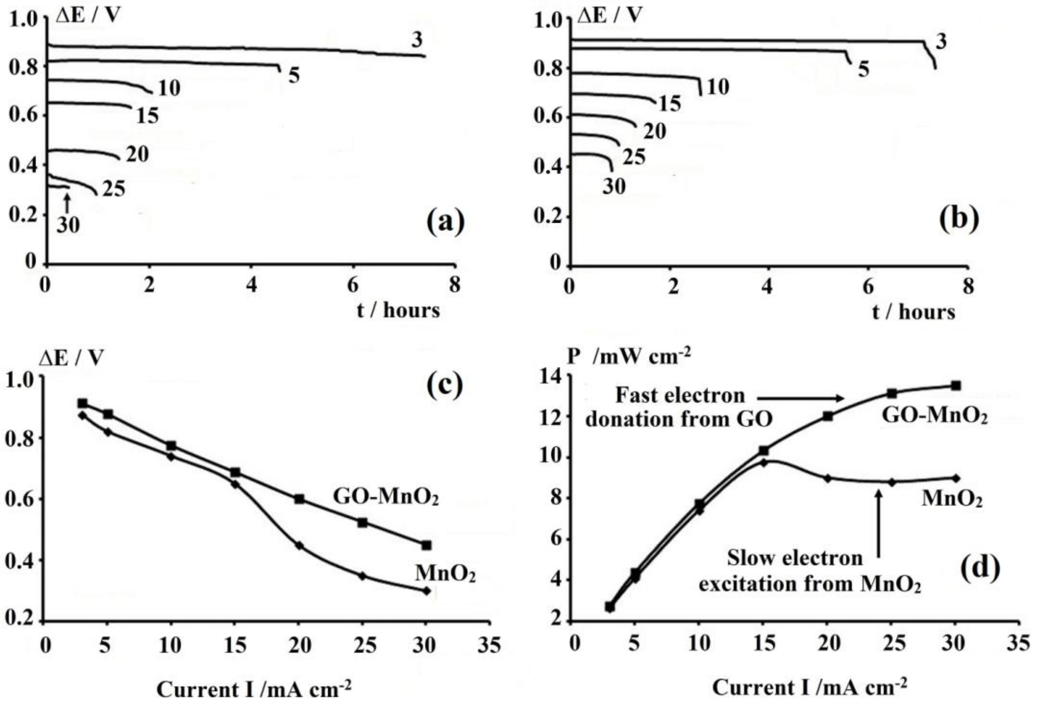

Figure SI-5. Chrono-potentiometry at various discharge currents for (a) Sn-MnO_2 cell (b) Sn-MnO_2/GO cell; (c) discharge potential ΔE/V vs. discharge current I/ mA cm^{-2} (d) plots of P/ mW cm^{-2} vs. I/ mA cm^{-2}.

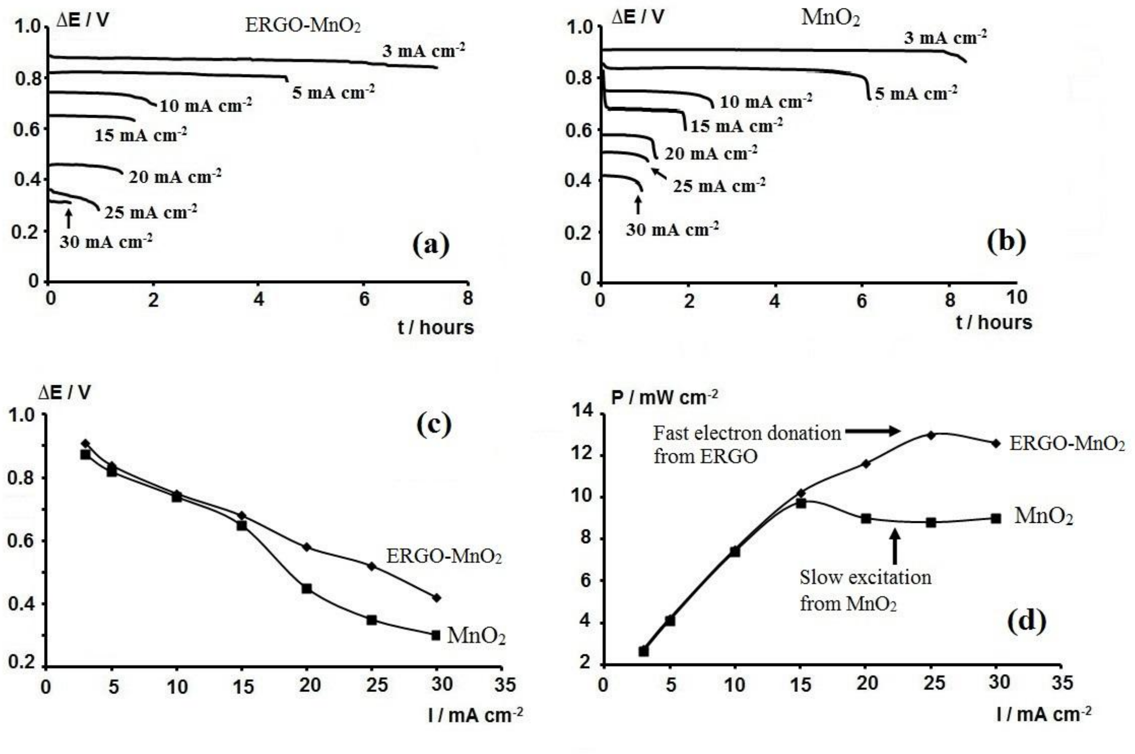

Figure SI-6. Chrono-potentiometry at various discharge currents for (a) Sn-MnO_2 cell (b) Sn-MnO_2/ERGO cell; (c) discharge potential ΔE/V vs. discharge current I/ mA cm^{-2} (d) plots of P/ mW cm^{-2} vs. I/ mA cm^{-2}.

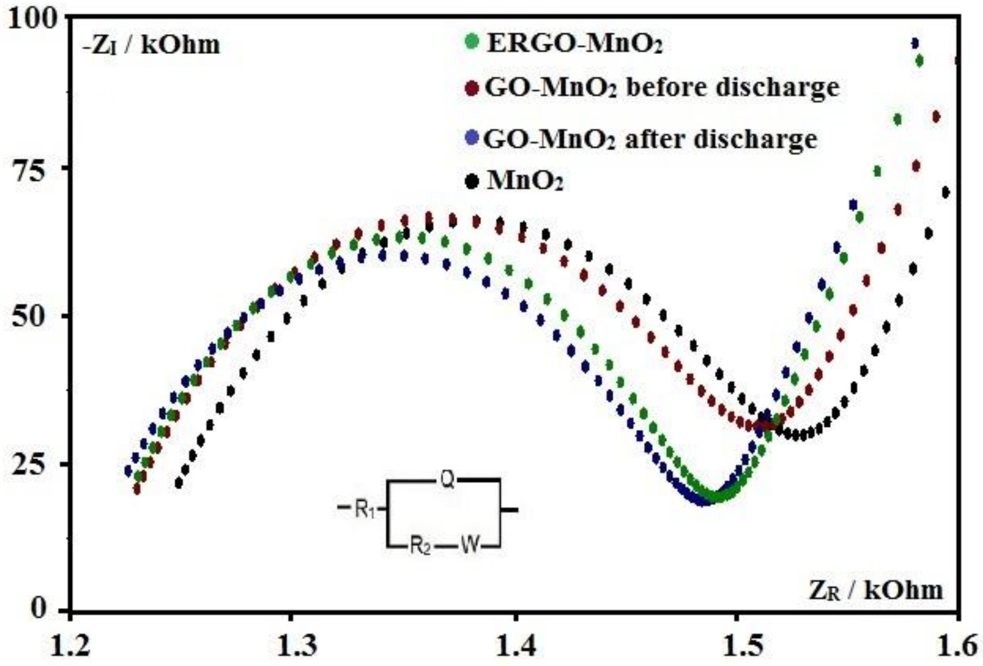

Figure SI-7. Nyquist plot in 6M KOH of MnO_2, GO-MnO_2 before discharge, GO-MnO_2 after discharge and ERGO-MnO_2.

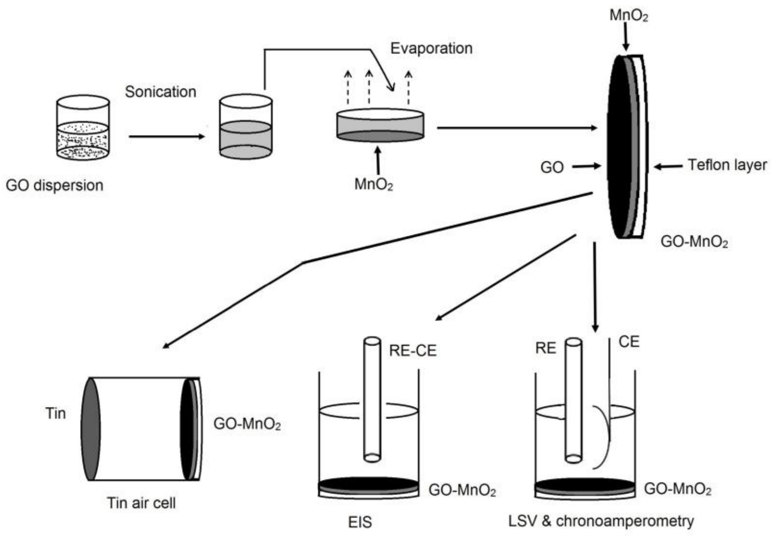

Figure SI-8 Summary of experimental procedures for the preparation of GO-MnO_2.

Table SI-1 Comparison of power density from different types of MnO_2 graphene composite air cathode from this work and previous works.

**Application**	**Power density / mW m^{-2}**	**Air cathode**	**Medium**	**Ref.**
MFC	2083	MnO_2-graphene nanosheet composite	Phosphate buffer solution, pH 7	11
	1470	MnO_2		
	1714	Pt/C		
MFC	3359	MnO_2 nanotube-GO composite	Phosphate buffer solution, pH 7	12
	1850	MnO_2 nanorods		
	2100	MnO_2 nanotubes		
Tin-air cell	13.0 x 10^4	GO-MnO_2	6M KOH	This work
	13.4 x 10^4	ERGO- MnO_2		
	9.2 x 10^4	MnO_2		

Table SI-2 Comparisons of the charge transfer resistance R_{CT} from this work and previous work.

**Application**	**Air cathode**	**Medium**	**R_{CT} / Ω cm^2**	**Ref.**
MFC	MnO_2-graphene nanosheets	Phosphate buffer solution, pH 7	124	1
	Pt/C		164	
	MnO_2		292	
Tin-air cell	MnO_2	6M KOH	197.2	This work
	GO-MnO_2		196.6	
	GO-MnO_2, after discharge		187.4	
	ERGO-MnO_2		187.0	
